# Hospital at home – a review of our experience

**DOI:** 10.1051/sicotj/2017047

**Published:** 2017-10-18

**Authors:** Edmond C.Y. U, Glyn A. Pryor, Martyn J. Parker

**Affiliations:** 1 Royal Stoke University Hospital Newcastle Road, Stoke-On-Trent ST4 6QG UK; 2 Peterborough City Hospital Peterborough PE3 9GZ UK

**Keywords:** Hip fracture, Rehabilitation, Early discharge, Hospital at home

## Abstract

*Introduction*: Hospital at home (HAH) is a service that provides home-based nursing and rehabilitation services whose aim is to prevent admission or to facilitate early discharge from care in an acute hospital.

*Methods*: We evaluated the effectiveness of early discharge hospital at home (HAH) schemes for hip fracture patients over a 27-year period in a district general hospital in the United Kingdom. A long-term database for audit and research purposes is maintained for all hip fracture patients admitted to Peterborough City Hospital. The data were analysed retrospectively and patients were followed up routinely for six weeks after discharge.

*Results*: As many as 8876 patients were admitted with a hip fracture between 1st January 1987 and 31st December 2014, of which 5512 patients were eligible for one of the two available HAH schemes. The proportion of eligible patients discharged to the HAH schemes, and their hospital stay and readmission rates were measured; 1786 patients were discharged to a HAH scheme. The proportion of patients discharged to the scheme progressively reduced from a maximum of 94% to a minimum of 13% over the study period. The length of hospital stay until discharge to the scheme progressively increased from a mean of eight days to 18 days.

*Discussion*: We conclude that HAH schemes can potentially reduce the length of hospital stay of hip fracture patients but continued resources and service organisation have to be provided to match the increasing demand to prevent the service from becoming ineffective.

## Introduction

Hospital at home (HAH) is a service that provides home-based nursing and rehabilitation services [1, 2] whose aim is to prevent admission or to facilitate early discharge from care in an acute hospital [3, 4]. The concept of HAH originated from France as “Hospitalisation à Domicile,” with the premise that inpatient hospital care is more costly and not all inpatients require the range of facilities and services that an acute hospital provides. Some patients would also prefer to be treated in their own home rather than in hospital.

It has been shown that HAH schemes are more cost efficient than care in the acute hospital [5]. Consequently, such schemes are a focus of interest for health strategists trying to create a primary care-led NHS [6]. These schemes have proven to be particularly relevant to the treatment and rehabilitation of patients with fractured neck of femurs, the incidence of which has been increasing over recent decades [7]. These patients occupy acute orthopaedic beds beyond their medical requirement; 51% of patient days were spent recovering from surgery without complications, and a further 28% were spent awaiting discharge after acute medical and surgical care had been completed [8].

Peterborough was one of the first areas in the United Kingdom to instigate a HAH scheme in 1978 [9]. Two separate HAH schemes were running for patients admitted to Peterborough City Hospital depending on the catchment area they resided in. The aim of this paper was to review our experience of these two HAH schemes utilised by Peterborough City Hospital over the past 27 years. We focus on trends in the uptake of the service and their consequent impact on acute orthopaedic bed use.

## Methods

The Peterborough HAH scheme consists of a multidisciplinary team that provides care to convalescing hip fracture patients in the patient’s own home [9]. The service provides care to patients selected to be suitable for the HAH scheme delivered by trained nurses, healthcare assistants, physiotherapists, and occupational therapists, all in the patient’s own home for up to 24 hours a day under the medical supervision of the general practitioner. Additionally, if required, the service can bring in social services, “meals on wheels” and home help. The amount of care is tailored to the individual patient’s requirements, and the scheme is generally continued for up to two weeks. If necessary, other community services take over from the scheme. This scheme covered the city of Peterborough and areas to the south of the city. The South Lincolnshire HAH scheme was started in 1999 using exactly the same principles and this covered the towns of Stamford, Bourne and the surrounding villages.

A long-term audit and research database has been maintained for all hip fracture patients admitted to Peterborough City Hospital since 1987. The data were collected and recorded prospectively as the patient was admitted, on a standard proforma which includes patient demographics, functional, social, and mental status, type of fracture (intracapsular versus extracapsular), surgical outcome, total hospital stay and discharge destination. The use of community resources on discharge was also recorded. The patients were followed up routinely in a dedicated hip fracture clinic six weeks from discharge whereby further data were collected on any complications experienced, any readmissions and whether there was any change in functional and social status. The final follow-up was at one year by review in clinic or telephone assessment. Patient deaths were also recorded in the database.

## Results

For the period 1 January 1987–31 December 2014, 8876 consecutive patients were admitted with a fractured neck of femur to Peterborough City Hospital. Only those patients who were eligible for the HAH schemes were included in this study; namely those who were admitted with hip fracture and lived in their own home, rented accommodation at home or warden-controlled accommodation. We therefore excluded those patients admitted from institutional care and those patients who fell in hospital causing the fracture. Patients from their own homes and outside the catchment area of the two schemes were also excluded, as were those from the South Lincolnshire HAH catchment area before the scheme started in 1999. In total for this time period, 5512 patients were eligible for the HAH schemes and therefore included in this study.

The characteristics of the patients are shown in Table 1. Both the Peterborough HAH scheme and the South Lincolnshire HAH scheme cover very similar patient demographics in terms of mean age, the high predominance of female patients and their social circumstances. Surgical outcomes were also recorded for these patients and there were no differences in their treatment methods. The surgical outcomes for the two groups of patients were also very similar. However, the surgical outcome is beyond the scope of this study and results have been previously published [10].

**Table 1.** Patient characteristics (%).

	Peterborough HAH catchment area patients	South Lincolnshire HAH catchment area patients
Time period assessed	1.1.1987 to 31.12.2014	1.1.1999 to 31.12.2014
Number of patients	4626	886
Discharged to HAH score	1476 (31.9%)	310 (35.0%)
Mean age [range]	77.2 [15–104]	79.6 [25–100]
Number of female	3467 (75.1%)	673 (76.0%)
Mental test score (out of 10)	6.6	7.3
ASA grade 1 or 2	1738 (37.6%)	370 (41.8%)
Mean ASA grade	2.6	2.6
Living alone	2534 (54.8%)	443 (50.0%)
Used walking aids in the house	1856 (40.1%)	373 (42.1%)
Intracapsular fracture	2687 (58.1%)	532 (60.0%)
Arthroplasty	1480 (31.3%)	297 (33.5%)
Extramedullary fixation of trochanteric fracture	1387 (30.0%)	226 (25.5%)
Intramedullay nail	909 (19.6%)	275 (31.0%)
Screw fixation of intracapsular fracture	774 (16.7%)	80 (9.0%)
Conservative	76 (1.6%)	8 (0.9%)

**Figure 1.**
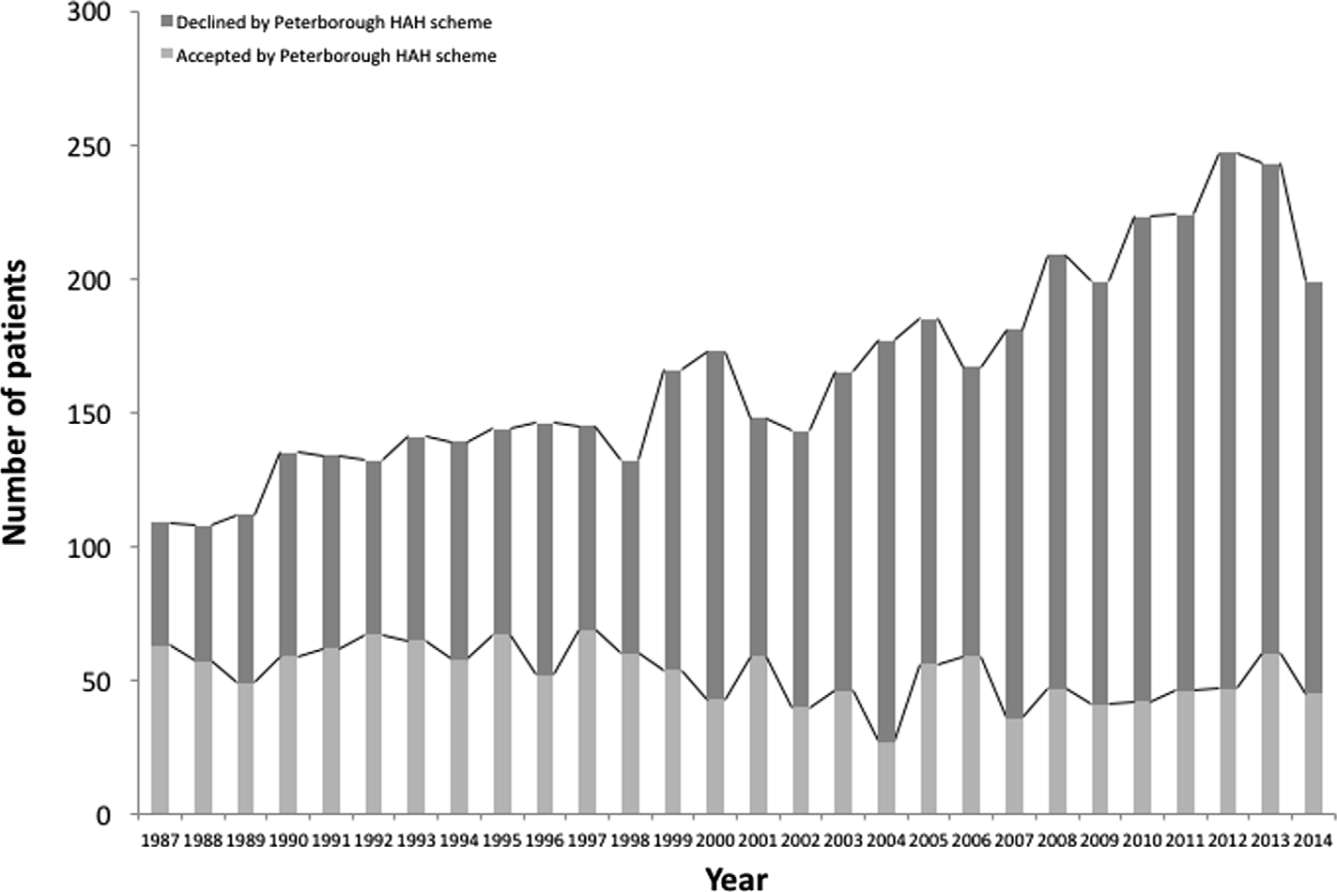
The number of patients accepted and declined by the Peterborough HAH scheme.

**Figure 2.**
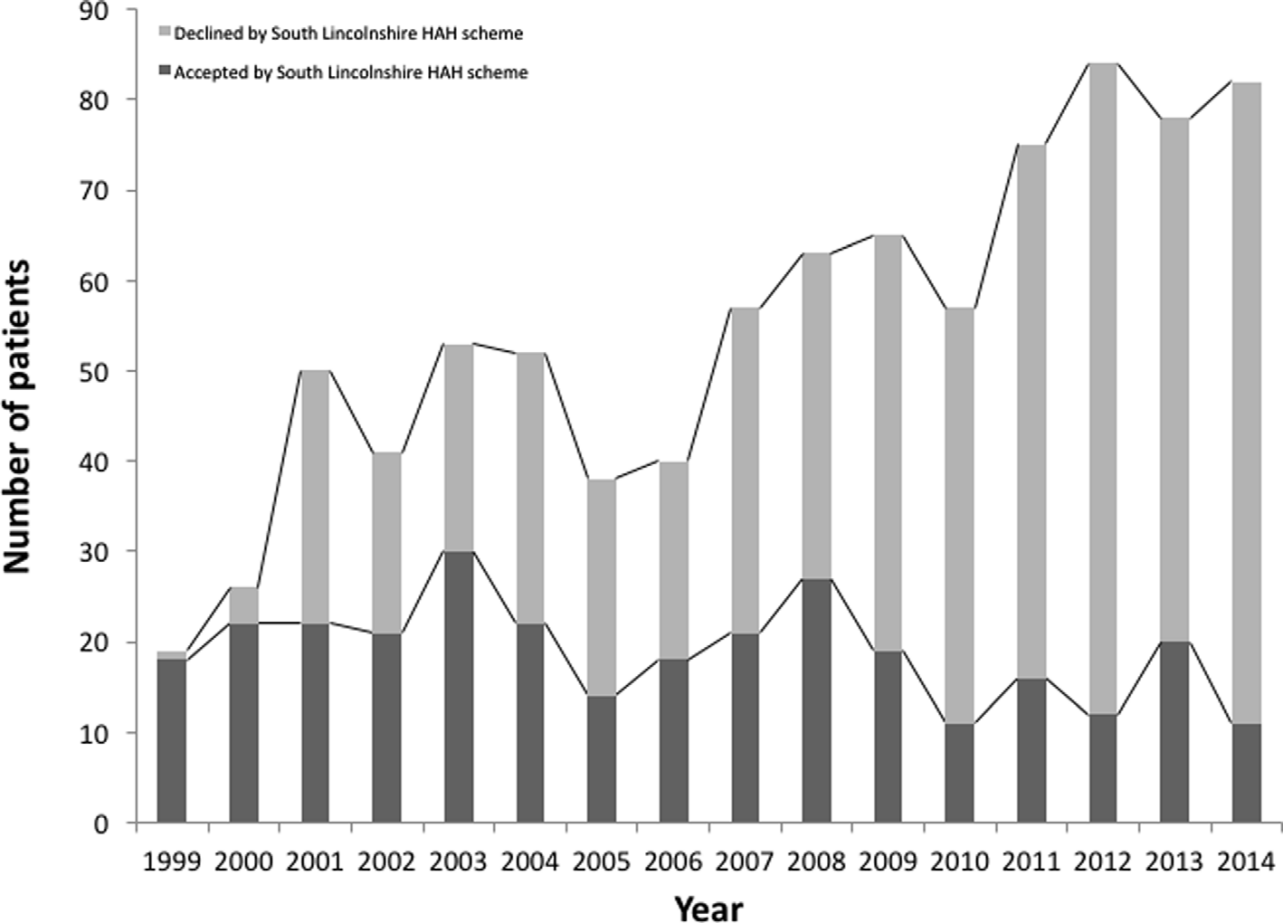
The number of patients accepted and declined by the South Lincolnshire HAH scheme.

Figures 1 and 2 detail the number of patients accepted and declined by the Peterborough HAH and the South Lincolnshire schemes. In the early years of the schemes, approximately 50% of eligible patients were discharged to the Peterborough HAH scheme and over 80% to the Lincolnshire scheme. These percentages fell to about 20% by the end of the study period. The table also details the increased number of patients treated over the study periods; for the Peterborough HAH scheme, the number of eligible patients rose from 109 in 1987 to 199 in 2014. Similarly, for the South Lincolnshire HAH scheme, the number of eligible patients rose from 19 in 1999 to 82 in 2014. This is consistent with the increase in number of patients admitted with a hip fracture – from 209 in 1987 to 360 in 2014 with a peak of 473 patients in 2012.

**Figure 3.**
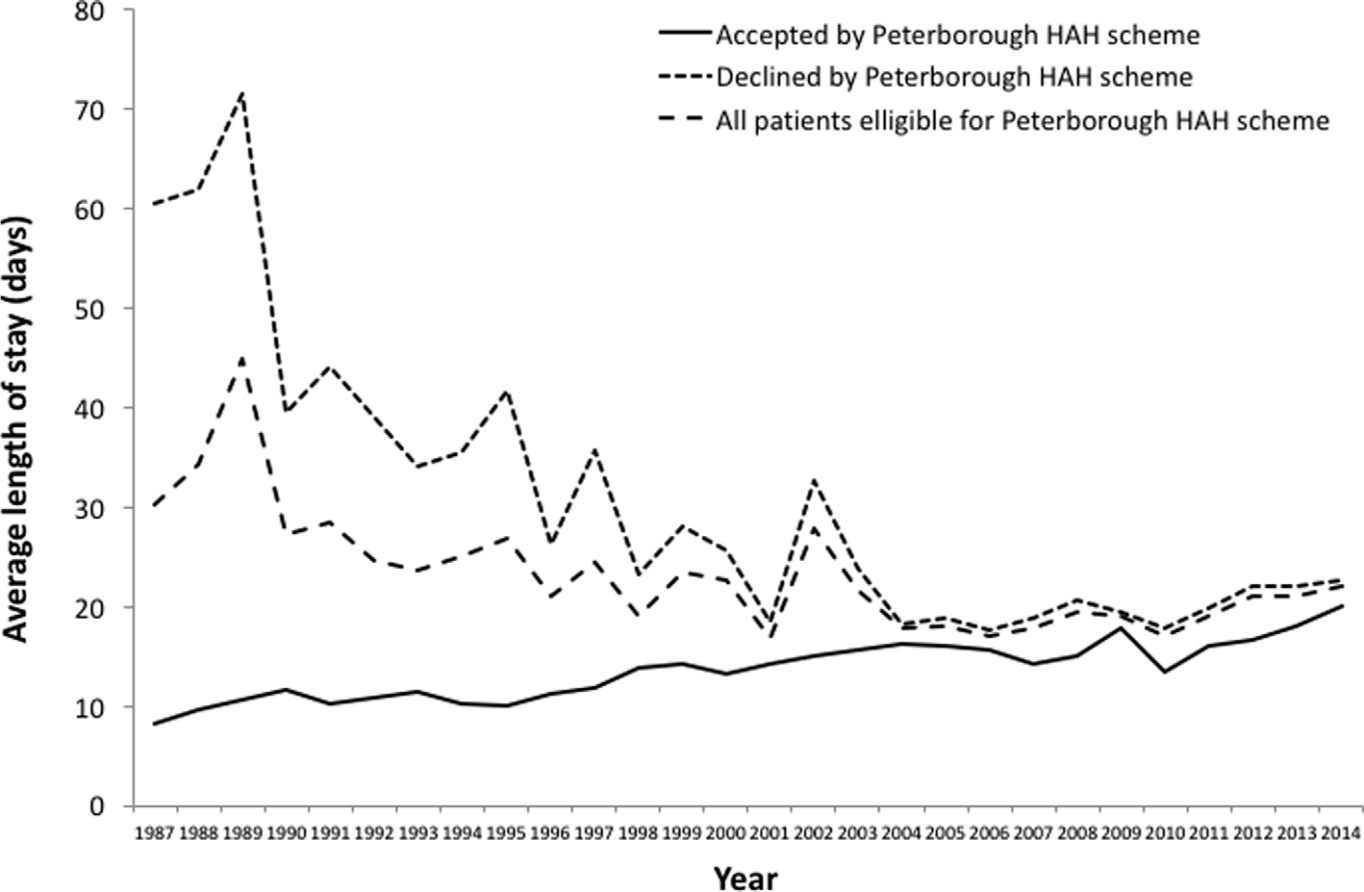
The length of stay of all patients within the catchment area of the Peterborough HAH scheme, and those accepted and declined by the scheme.

**Figure 4.**
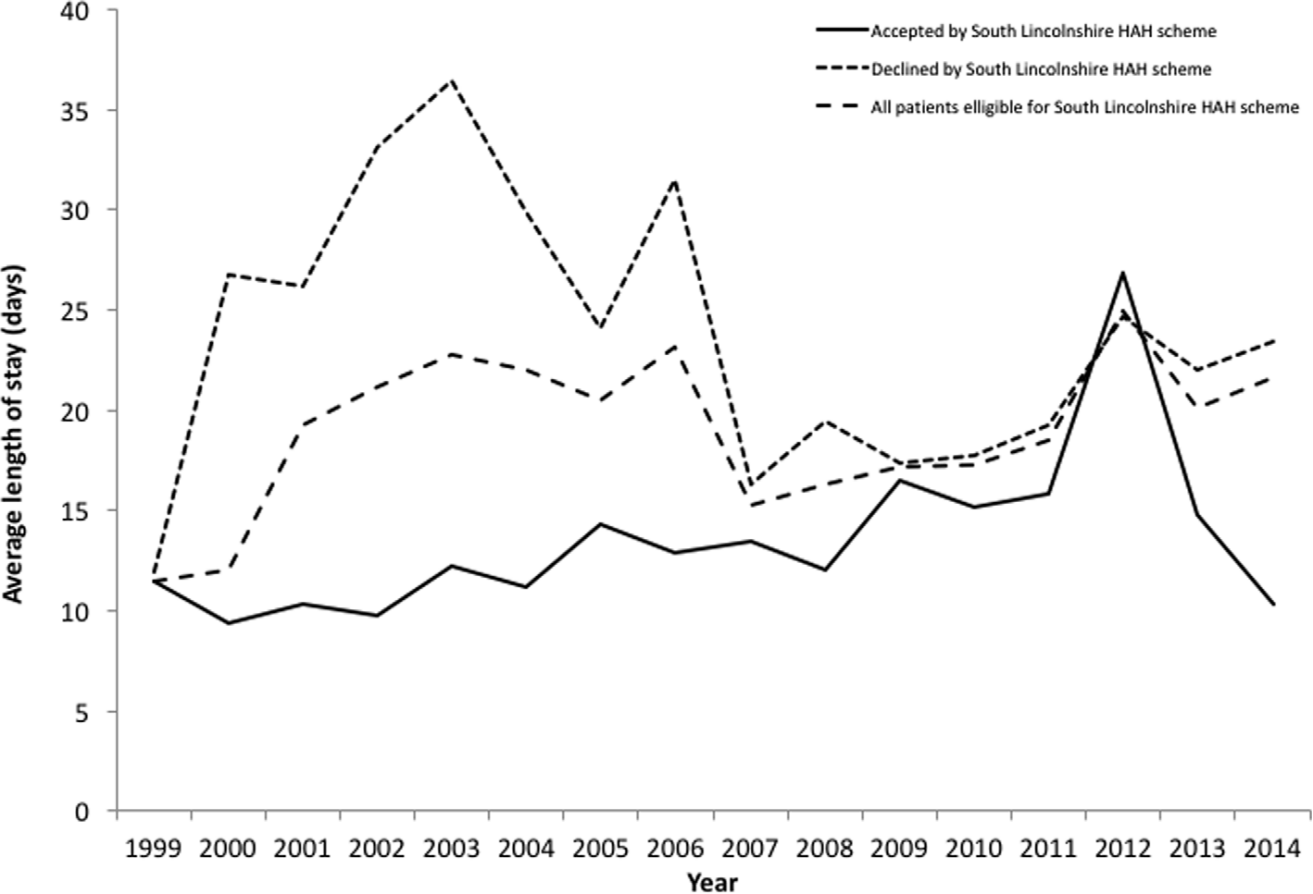
The length of stay of all patients within the catchment area of the South Lincolnshire HAH scheme, and those accepted and declined by the scheme.

Figures 3 and 4 detail the average hospital stay for the two schemes dependent on whether the patient was admitted or not to the HAH scheme. The average hospital stay for all eligible patients is also given.

**Figure 5.**
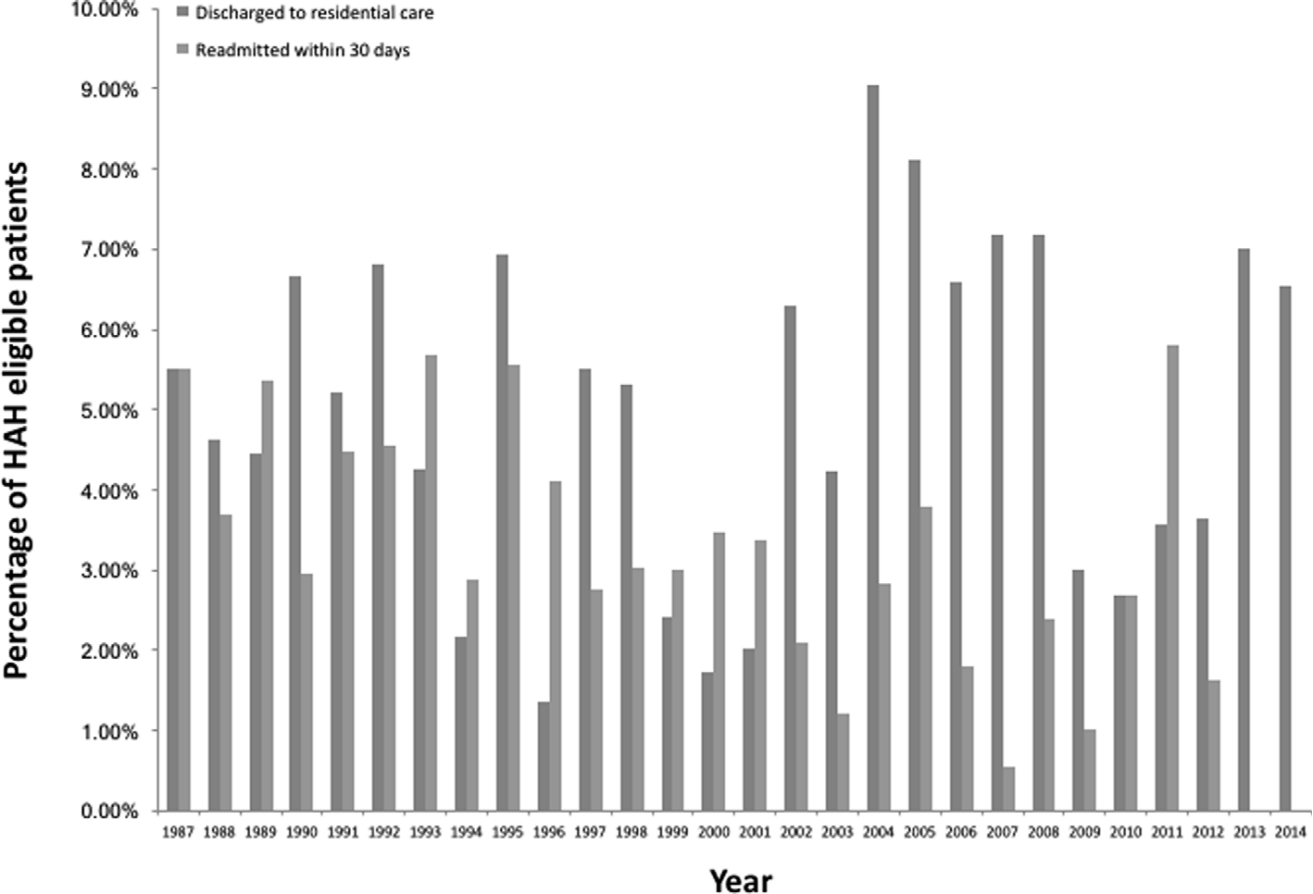
The percentage of patients discharged via Peterborough HAH scheme who were discharged to residential care and readmitted within 30 days.

**Figure 6.**
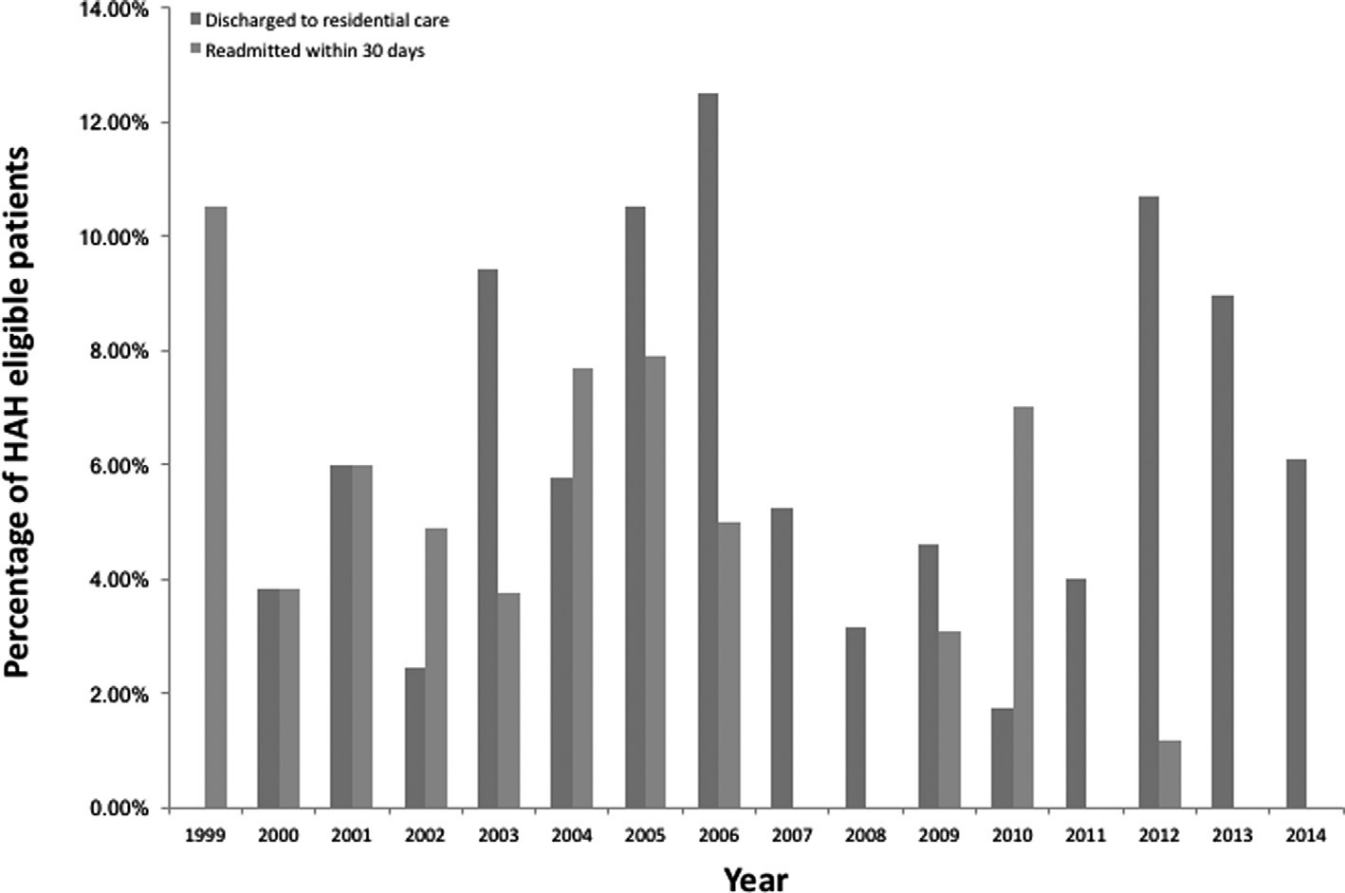
The percentage of patients discharged via South Lincolnshire HAH scheme who were discharged to residential care and readmitted within 30 days.

Figures 5 and 6 detail the percentage readmission rates and percentage of patients who were not able to be discharged back home and required permanent placement in institutional care. The readmission rate was for 30 days from discharge and only for reasons related to the hip fracture, which included problems with rehabilitation, medical and surgical complications. These indicate there were no notable differences in the readmission rate or need for institutional care over the years, between patients accepted and declined by HAH schemes.

## Discussion

Over the past 27 years the number of patients with hip fractures treated at Peterborough City Hospital has increased. This is consistent with the rising incidence of hip fractures globally – largely a result of the ageing population [7, 11]. At the start of the Peterborough HAH scheme in 1987, 57.8% of patients with hip fractures eligible for the scheme were accepted, however by 2014 only 22.61% were accepted (Figure 1). This is a similar scenario for the Lincolnshire HAH scheme; at its start in 1999, 94.7% of eligible hip fracture patients were accepted but by 2014, only 13.4% were accepted (Figure 2). Overall the number of patients accepted by each scheme has remained relatively similar over the years. The rising number of patients treated for hip fractures has not been matched by a rise in HAH scheme resources, leading to a gradual decline in the percentage of patients being accepted onto the schemes.

At their conception, the HAH schemes showed promising results at Peterborough City Hospital with regard to reducing the average length of in hospital stay. In 1987 at the start of the Peterborough HAH scheme, the average length of stay of hip fracture patients accepted onto the scheme was about seven times lower than that of those declined by the scheme (Figure 3). Similarly, in 2000 near the start of the Lincolnshire HAH scheme, the average length of stay of patients accepted onto the scheme was nearly three times less than that of patients declined (Figure 4). The average length of stay of patients accepted onto the Peterborough and Lincolnshire HAH schemes has risen slowly since their respective starts in 1987 and 1999 due to the increasing number of patients eligible for the schemes without a matched increase in the provision of HAH resources – this means that patients are having to wait longer in hospital after surgery before they are discharged via the scheme (Figures 3 and 4).

However, there has also been a large decline in the average length of stay of those eligible patients who were declined by the Peterborough HAH scheme. Over the past 27 years, the average length of stay of patients declined by the Peterborough HAH scheme has fallen by approximately two thirds (Figure 3). This has been due to advances in the provision of services from admission through A&E to discharge with social services, a multidisciplinary approach involving therapists and nursing staff, and also improvements in fracture and medical management [12, 13]. Whilst large differences in the average length of stay existed for patients in the Lincolnshire HAH catchment area between 2000 and 2006, these differences have become marginal from 2009 onwards (Figure 4).

Our experience from the Peterborough and Lincolnshire HAH schemes is that such schemes have no effect on the proportion of patients requiring institutional care in a residential home or any effect on the proportion of patients requiring readmission from complications (Figures 5 and 6). This indicates that HAH schemes do not encourage the inappropriate early discharge of patients from hospital and that such patients also receive appropriate rehabilitation within their own home enabling to continue residing there.

Previous studies have shown HAH schemes to be more cost-effective when compared with acute hospital care [5]. The main determinant of the cost of treatment for a hip fracture is the length of institutional stay [14]. Lawrence et al. showed the mean total hospital expenditure per patient was calculated to be 12,163 pounds sterling of which ward costs accounted for 84% [14]. With the assistance of a health economist, we undertook a cost analysis of early discharge with HAH schemes and found that they produced savings of 722 pounds sterling per patient at 1991/2 prices (1384.50 pounds sterling at current prices) [5]. As such, expansion of HAH schemes to cope with the demand of eligible patients could lead to cost savings for the NHS by facilitating their earlier discharge from more expensive acute hospital care.

The limitation of our study is that, it is difficult to make any direct inference of the impact of HAH schemes on the average length of stay, mortality and readmission rates. As HAH patients are generally in a state of better health, this could also have a large influence on these factors. Other factors that were not measured include the precise social circumstances of individual patients as often complex social arrangements can impede upon discharge and consequently their ability to utilise HAH schemes.

To our knowledge, there are no other studies looking at the impact of HAH schemes on such a large group of patients with hip fractures, and its influence on their length of hospital stay and its relationship with morbidity and mortality. Since the inception of HAH schemes in 1978, few studies have reflected on their role in the management of patients with hip fractures despite several analyses promoting their cost benefit over inpatient rehabilitation [5].

In conclusion, despite a rising incidence of hip fractures, there has not been a matched rise in the provision of HAH resources to treat these patients within our catchment population.

## Conflict of interest

All authors declare no support from any organisation for the submitted work; no financial relationships with any organisations that might have an interest in the submitted work; no other relationships or activities that could appear to have influence the submitted work.

The authors have followed the STROBE checklist in collecting and reporting their data. Elements of the checklist are incorporated into the manuscript.

Ethical approval: Not needed.

Funding: None.
